# Outcomes of Surgical Bed Versus Tumor Margins in Trans-Oral Resection of Early Glottic Cancer

**DOI:** 10.1007/s12070-025-05469-6

**Published:** 2025-04-24

**Authors:** Tom Jacob, Narin Nard Carmel Neiderman, Yotam Lior, Anton Warshavsky, Gilad Horowitz, Oshri Wasserzug, Leonore Trejo, Nidal Muhanna, Yael Oestreicher-Kedem

**Affiliations:** 1 Department of Otolaryngology, Head and Neck Surgery and Maxillofacial Surgery, Tel Aviv Sourasky Medical Center, Affiliated to the Faculty of Medical and Health Sciences, Tel Aviv University, 6 Weizmann St, Tel Aviv, 6423906 Israel; 2 Division of Anesthesia, Intensive Care and Pain Management, Tel Aviv Sourasky Medical Center, Affiliated to the Faculty of Medical and Health Sciences, Tel Aviv University,, Tel Aviv, Israel; 3 Institute of Pathology, Tel Aviv Sourasky Medical Center, Affiliated to the Faculty of Medical and Health Sciences, Tel Aviv University, Tel Aviv, Israel

**Keywords:** Larynx, Cancer, Margin, Cordectomy, Frozen section

## Abstract

To assess which margin status, surgical bed margin (SBM) or tumor margin (TM) correlates best with outcome following transoral laser microsurgery (TLM) for early (Tis-T2N0M0) glottic cancer. A retrospective cohort study including patients with early (T1-2) glottic cancer. Data on TM status, SBM status, recurrence rate, and disease-free survival (DFS) were retrieved from the medical records of all patients who underwent vocal fold (VF) cordectomy due to Tis-T2N0M0 glottic squamous cell carcinoma from January 2013 to February 2021. Only patients with available data on both SBM and TM status were included in the study. Forty patients, 34 (85%) with disease-free SBM and TM, and 6 (15%) with disease-free SBM but involved TM, were included. Four (10%) patients developed recurrence, all in the group of both disease-free SBM and TM. The recurrence, 2-year disease free survival and survival at the end of follow-up (median 37.5 months) rates were 11%, 94.1% and 97.1% and 0%, 100% and 100%, in the groups of disease-free SBM and TM and disease-free SBM but involved TM, respectively. There were no statistically significant group differences. TM involvement, in the presence of disease-free SBM, did not compromise outcome.

## Introduction

The aim of TLM in early glottic cancer is to obtain local control (LC) and achieve cure and prolong survival while maintaining laryngeal function [1–3]. Preservation of maximal laryngeal function is mostly achieved by complete tumor resection with the narrowest free margins to preserve as much healthy and functioning laryngeal tissue as possible [4]. While there is no doubt that preservation of function is a highly important goal, it must go hand in hand with a low recurrence rate and a high LC rate.

Some studies suggest that disease-free margins have a direct impact upon prognosis and recurrence rates [3, 5, 6], while other suggest that those margins do not correlate with LC rates [3, 7–10], specifically when referring to a single positive margin or superficial margins [2, 11]. Moreover, concerns about specimen size resulting from piecemeal removal techniques and the complex orientation in the small and resected specimen makes it difficult to characterize margin status [12].

To date, there is no consensus about the preferred method of evaluating margin status during and after TLM in early glottic cancer. One of the available techniques is frozen section (FS) analysis of the surgical bed margin (SBM) during surgery followed by intraoperative extension of the resection area if needed, and it is a commonly used method to achieve better LC rates and avoid the need for revision surgery [13]. However, some studies claim that performing FSs of the SBM samples damages the specimens which are intended for definitive pathologic examination, and that it also prolongs the operating time [12]. A SBM permanent biopsy is another way to determine tumor margin status. SBM biopsies can help detect patients at risk of recurrence, improve the LC rate, and lower the incidence of unnecessary SL procedures [4, 14]. It should be noted that SBM biopsies had not been routinely taken in those studies, and that margin status did not have a significant influence on 5-year survival.

The aim of our study was to compare SBM status and TM status outcomes and to assess which margin status correlated best with LC and disease-free survival (DFS).

## Materials and Methods

### Study Population

This is a retrospective cohort study conducted at the Tel Aviv Sourasky Medical Center, a tertiary referral center. The study was approved by the institutional Helsinki committee (TLV-0902-20). Included were patients who underwent VF cordectomy due to Tis-T2N0M0 glottic SCC from January 2013 to February 2021 by one surgeon. Only patients with available histology data on both SBMs and tumor margins (TMs) were included.

### Outcome Measures

The patients’ medical records were reviewed. Data were retrieved on their demographics, medical history, tumor location and stage, oncologic treatments and surgical procedures, follow-up, recurrence, disease status at last follow-up and histology results. Potential correlations between FS SBM status, permanent section TM status, permanent section SBM status, LC recurrence, and DFS were also evaluated.

### Management Approach

The diagnosis of VF malignancy was determined either by in-office, awake, transnasal flexible fiberoptic laryngeal biopsy [20], or by direct microscopic laryngoscopy (DML) and biopsy (incisional or excisional) under general anesthesia. Once the diagnosis of early VF malignancy had been histologically established, treatment options (cordectomy versus radiotherapy) were discussed with the patient. Patients, who elected to undergo surgery, underwent TLM and cordectomy. The type of cordectomy was determined in accordance with tumor extent and the surgeon’s clinical judgment. All operations were performed by means of a CO_2_ laser (Luminis Sharplan 40c CO_2_ or Alma Pixel CO_2_).

The tumors were resected en-bloc in most cases and marked with sutures for border orientation. Larger tumors were resected in two or three pieces. Each piece was oriented and marked for pathology analysis. These borders are defined in our study as tumor margins. Representing sample biopsies from five different areas (anterior, posterior, superior, inferior and deep) from the surgical bed were taken and sent for FS analysis. These biopsies represent in our study SBM. In cases of an intraoperatively detected involved FS border, the resection on the involved border was extended and the last resection of this border area was considered the SBM. Permanent section margin status was determined after paraffin and H&E stain of both the SBM and the TM.

The patients were regularly followed with flexible or rigid fiberoptic laryngoscopy with expending gaps, up to every 6 months during the fifth year after cordectomy, and yearly afterwards. Suspicious lesions were biopsied.

### Statistical Analysis

Continuous variables were evaluated for normal distribution with histograms and reported as mean and standard deviation (normally distributed variables) or median and interquartile range (skewed variables). Categorical variables were reported as frequency and percentage. The chi-square test or Fisher’s exact test were used to compare categorical variables between the two groups of patients, and the independent samples T test or Mann–Whitney test was used to compare the continuous variables. *P* <.05 was considered as statistically significant.

## Results

### Demographics and Staging

Forty patients (36 males, 4 females, mean age 71.83 years, range 44–89 years) with Tis-T2 glottic squamous cell carcinomas (SCCs) and available data on both the SBMs and the TMs comprised the study cohort (Table 1). TLM cordectomy was the initial treatment of their glottic malignancy in 37 of the 40 patients and three underwent TLM cordectomy for recurrence (two after radiotherapy and one after initial superficial cordectomy [elsewhere]). The cordectomy types were Type 1 (*n* = 1), Type 2 (*n* = 1), Type 3 (*n* = 35), and Type 5 (*n* = 3).

**Table 1:** Demographics and tumor parameters of the study cohort (*N* = 40)

Number of patients	40
Mean age, y (range)	71.83 (44–89)
Male	36 (90%)
Smoker	36 (90%)
Histology	
Carcinoma in situ	9 (22.5%)
Squamous cell carcinoma	29 (97.5%)
Verrucous carcinoma	1 (2.5%)
T stage	
In situ	9 (22.5%)
T1	23 (57.5%)
T2	8 (20%)
Involved frozen section margin leading to intraoperative extension of resection in the area of involved margin	4 (10%)
Disease-involved permanent section tumor margin	6 (15%)
Disease-involved permanent section surgical bed margin	0
Recurrence	4 (10%)
Two-year disease-free survival	38 (95%)
Disease free at last follow-up	39 (97.5%)
Mean follow-up in months (range)	37.5 (2–91)

### Intraoperative Frozen Section Margins

Intraoperative FSs of the SBMs were taken in 36 patients (31 had later disease-free SBM and TMs in permanent section and in 5 had later disease-free SBM but disease-involved TMs in permanent section) (Table 2). In the group of permanent section disease-free SBM and TMs with available intraoperative FS (*n* = 31), the initial SBM FS was positive for malignancy in two (5.3%) patients: one of them had two involved SBMs and one had one involved SBM. Both patients underwent intraoperative extension of the resection in the areas of disease-involved margins, so all permanent section SBM FSs were disease-free. In the group of permanent section disease-free SBM but permanent section disease-involved TMs with available intraoperative FS (*n* = 6), the FS was positive in two. One patient had two disease-involved margins and one had one disease-involved margin. Both patients underwent intraoperative extension of the resection in the areas of the disease-involved margins, so all permanent section SBM FSs were disease-free.

**Table 2:** Comparison of the group with Disease-free SBMs and TMs and the group with Disease-free SBMs and Disease-involved TMs

	Free SBMs and Free TMs	Free SBMs and involved TMs	*P*-value
Male	30 (88.2%)	6 (100%)	1
Smoker	29 (85.3%)	4 (66.7%)	1
T stage			
Tis-1	28 (82.4%)	4 (66.7%)	0.58
T2	6 (17.6%)	2 (33.3%)	
Frozen section margin requiring intraoperative extension of resection in the area of a disease-involved margin	2 (5.3%)	2 (33%)	0.01
Recurrence (*n* = 40)	4 (11.8%)	0	1
Two-year disease-free survival (*n* = 32)	26 (*n* = 28, 92.9%)	4 (*n* = 4, 100%)	1
Five-year disease-free survival (*n* = 11)	8 (*n* = 10, 80%)	1 (*n* = 1, 100%)	1
Disease free at last follow-up (*n* = 40)	33 (*n* = 34, 97.1%)	6 (*n* = 6, 100%)	1

SBM, surgical bed margin; TM, tumor margin

### Permanent Section Margin Involvement

All 40 permanent section SBMs were negative for malignancy on the pathologic assessment: 32 patients’ margins that were initially negative on FS, 4 patients’ permanent section FS margins after re-resection due to initial disease involved FS and the 4 patients’ SBM margins that were not sent for FS.

Thirty-four (85%) patients had negative TMs in the permanent section pathology report, and six (15%) patients had disease-involved TMs. The 34 patients with both a negative SBM and a negative TM included 30 (88.2%) males, 29 (85.3%) patients who had a history of smoking, nine (26.5%) patients who were Tis, 19 (55.9%) patients who were T1, and six (17.6%) patients who were T2. The six patients with a negative SBM but a disease-involved permanent section TM were all males, four (66.7%) had a history of smoking, four (66.7%) were T1, and two (33.3%) were T2. There were no significant differences between the groups in age, sex, smoking history, or disease staging.

### Recurrence and Survival

The median follow-up time was 37.5 months (range 2–91), and 39 (97.5%) patients were free of disease at the last follow-up. Thirty-two patients had a follow-up of at least 2 years and the two-year DFS rate in this group was 93.7%. Eleven patients had a follow-up longer than 5 years and the five-year DFS rate in this group was 81.8%. Four (10%) patients (Tis and T1), all from the group of permanent section disease-free SBMs and TMs, developed recurrent glottic SCC at a median of 15.5 months (range 7–34 months). No recurrence was noted in the group of permanent section disease-free SBMs but disease-involved TMs. There were no significant study group differences in the two-year DFS (*n* = 32, 92.9% vs. 100%, respectively), in the five-year DFS (*n* = 11, 80% vs. 100%, respectively) or in the disease status at the last follow–up (97.1% vs. 100%) (Table 2).

Recurrences were treated by revision cordectomy (*n* = 1, initial Tis, recurrent TisN0M0), radiotherapy (*n* = 1, initial anterior commissure T1, recurrent T3N2M0), chemoradiotherapy (*n* = 1, initial T1, recurrent T3N1M1), or total laryngectomy (*n* = 1, initial T1, recurrent T4aN0M0). One of these patients (with recurrent T3N1M1) died of disease despite salvage treatment consisting of chemoradiation.

Four of our study patients had a disease-involved initial (rather than permanent section, which was the basis for dividing to groups) SBM and underwent extension of resection during primary cordectomy (two in the disease-involved TMs group and two in the disease-free TMs group). One of the four developed a recurrence (TisN0M0) which was treated by re-resection 8 months after the initial cordectomy and was free of tumor at last follow-up. No significant differences in recurrence rate and DFS were found between the group of patients with intraoperative re-resection of disease-involved SBM (*n* = 4) on FS and the group of initial disease-free SBM (*n* = 32) on FS.

## Discussion

The aim of the present study was to assess the significance of disease-free SBM versus disease-free TM in terms of local control and DFS in early (Tis-2) glottic cancer. The comparison revealed that the TM status did not significantly influence LC or DFS in the presence of disease-free permanent section SBMs. To note, our cohort was limited to patients with disease-free permanent section SBMs, because when involved margins were found on FS analysis during the cordectomy an extension of resection of this margin was performed.

In early glottic SCC it is debatable whether positive margins (either TM or SBM) are associated with worse prognosis [21, 22]. Jumaily et al. [22], did not find a negative impact of positive margins on overall survival among 747 patients with early-stage glottic cancer treated by TLM. Hendriksma et al. [4], found that disease-involved SBMs correlated with lower LC rates, while other studies [12, 22] did not find any correlations between disease-involved margins and LC or DFS. Although the official National Comprehensive Cancer Network^®^ guidelines [1] recommend achieving disease-free margins to avoid recurrence, there is no recommendation of which type of margin (SBM or TM) should be assessed to achieve the highest rates of LC and DFS. None of our study patients had permanent section disease-involved SBM since the patients with disease-involved SBMs detected by FS analysis during the cordectomy underwent intraoperative extension of resection in the area of the disease-involved margins, which is a common approach if margins are found to contain malignant cells on FS analysis during a primary cordectomy [23, 24]. Some studies suggest that disease-involved initial resection margins lower DFS rates, despite extension of the resection during primary cordectomy (rather than achieving a disease-free margin in the initial incision). Four of our study patients had a disease-involved initial intraoperative FS SBMs and underwent extension of the resection of those margins during primary cordectomy (two in the disease-involved TMs group and two in the disease-free TMs group). One of them later developed a recurrence which was treated by re-resection and was free of tumor at last follow-up. We found no significant correlation between performing an extension of the resection and a change in recurrence rate or DFS, unlike other studies [17, 18, 25] that reported a higher recurrence rate.

Three of the four patients with tumor recurrence in our cohort were diagnosed with an aggressive recurrent disease. Factors such as multiple laryngeal procedures for recurrent dyplastic lesions (*n* = 1) and tumor locations (anterior commissure [*n* = 1] and proximity to the vocal process [*n* = 1]) [26, 27] may have influenced disease recurrence and the late stage at recurrence diagnosis in these patients. These factors were not investigated in our study but should be pointed out.

The rate of disease-involved TM found in our study (15%) and the rates of LC and two-year DFS (90% and 95%, respectively) are compatible with those in the literature [8, 19, 22]. Thus, we conclude that examining TMs as well as SBMs are at least as good as the conventional approach (when SBM biopsies are not taken), unlike other studies [10, 24] that advocate against taking SBM biopsies, claiming that errors in SBM analysis and interpretation can lead to false negative results and influence recurrence.

Our study has limitations that bear mention. It is retrospective in design and includes a small patient cohort recruited over a relatively long period. For example, to reach a significant difference between the two-year DFS rates with a power of 80% and a significance of 5%, a sample size of about 220 patients is needed, among them at least 34 patients with positive TMs. Since we encountered only 6 patients with a positive TMs, it was difficult to draw any definitive conclusions about the recurrence rates for each approach. We do, however, believe our observations are important even at this stage. Moreover, since we did not include any patients with disease-involved SBMs (due to extension of the resection during the initial cordectomy), we could not assess the influence of SBM involvement on LC and DFS.

The follow-up period, in most patients, was less than the recommended five-year follow-up for glottic cancer. However, there were no significant differences in the DFS and disease status at last follow-up between the two study groups at two-years (32 patients) and at five-years (11 patients) of follow-up (92.9% and 100% vs. 80% and 100%).

The results of this study demonstrated that TM involvement, in the presence of a disease-free permanent section SBM, did not predispose to increased tumor recurrence nor did it decrease two-year DFS.
